# *catena*-Poly[[bis­[4-(dimethyl­amino)­pyridine-κ*N* ^1^]cobalt(II)]-di-μ-azido-κ^4^ *N* ^1^:*N* ^3^]

**DOI:** 10.1107/S1600536813005205

**Published:** 2013-02-28

**Authors:** Fatiha Guenifa, Ouahida Zeghouan, Nasreddine Hadjadj, Lamia Bendjeddou, Hocine Merazig

**Affiliations:** a Unité de Recherche de Chimie de l’Environnement et Moléculaire Structurale (CHEMS), Faculté des Sciences Exactes, Campus Chaabet Ersas, Université Constantine I, 25000 Constantine, Algeria

## Abstract

The title layered polymer, [Co(N_3_)_2_(C_7_H_10_N_2_)_2_]_*n*_, contains Co^II^, azide and 4-(dimethyl­amino)­pyridine (4-DMAP) species with site symmetries *m*2*m*, 2 and *m*, respectively. The Co^2+^ ion adopts an octa­hedral coordination geometry in which four N atoms from azide ligands lie in the equatorial plane and two 4-DMAP N atoms occupy the axial positions. The Co^II^ atoms are connected by two bridging azide ligands, resulting in a chain parallel to the *c* axis.

## Related literature

For applications of coordination polymers, see: Fujita *et al.* (1994 ▶); Hagrman *et al.* (1999 ▶); Hoskins & Robson (1990 ▶); Yaghi & Li (1995 ▶). For a related Cu complex, see: Dalai *et al.* (2002 ▶).
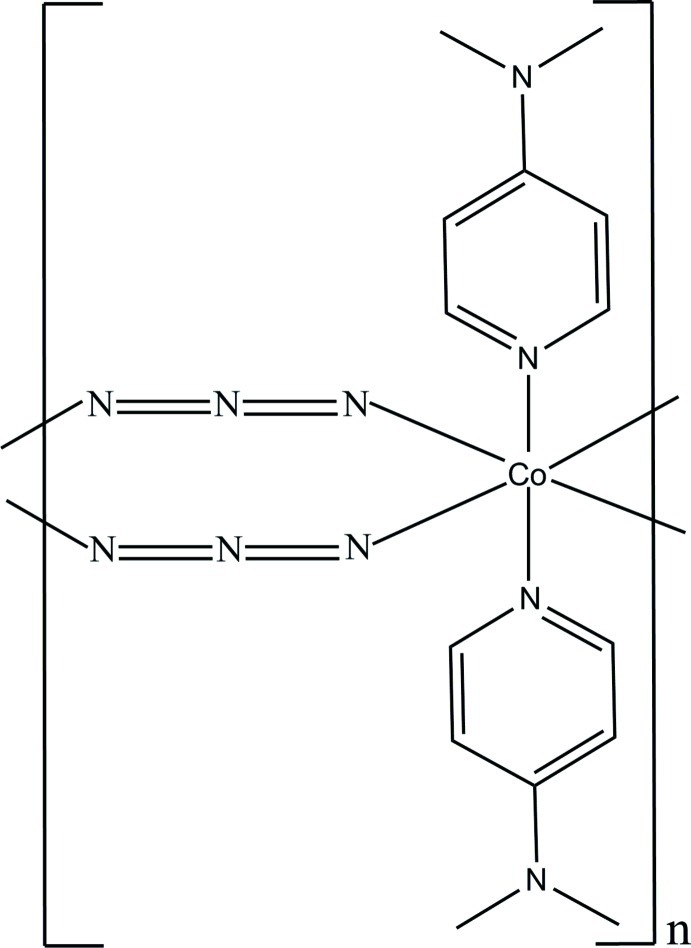


## Experimental

### 

#### Crystal data

[Co(N_3_)_2_(C_7_H_10_N_2_)_2_]
*M* *_r_* = 387.33
Orthorhombic, 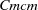

*a* = 9.622 (5) Å
*b* = 18.404 (5) Å
*c* = 9.734 (5) Å
*V* = 1723.7 (13) Å^3^
*Z* = 4
Mo *K*α radiation
μ = 1.02 mm^−1^
*T* = 293 K
0.1 × 0.09 × 0.08 mm

#### Data collection

Bruker APEXII diffractometer
5192 measured reflections
1393 independent reflections
1099 reflections with *I* > 2σ(*I*)
*R*_int_ = 0.031

#### Refinement

*R*[*F* ^2^ > 2σ(*F* ^2^)] = 0.032
*wR*(*F* ^2^) = 0.086
*S* = 1.07
1393 reflections
79 parameters
H-atom parameters constrained
Δρ_max_ = 0.43 e Å^−3^
Δρ_min_ = −0.37 e Å^−3^

### 

Data collection: *APEX2* (Bruker, 2006 ▶); cell refinement: *SAINT* (Bruker, 2006 ▶); data reduction: *SAINT*; program(s) used to solve structure: *SIR2002* (Burla *et al.*, 2003 ▶); program(s) used to refine structure: *SHELXL97* (Sheldrick, 2008 ▶); molecular graphics: *ORTEP-3 for Windows* (Farrugia, 2012 ▶); software used to prepare material for publication: *WinGX* (Farrugia, 2012 ▶), *Mercury* (Macrae *et al.*, 2006 ▶) and *POVRay* (Persistence of Vision Team, 2004 ▶).

## Supplementary Material

Crystal structure: contains datablock(s) global, I. DOI: 10.1107/S1600536813005205/vm2189sup1.cif

Structure factors: contains datablock(s) I. DOI: 10.1107/S1600536813005205/vm2189Isup2.hkl

Additional supplementary materials:  crystallographic information; 3D view; checkCIF report

## Figures and Tables

**Table 1:** Selected bond lengths (Å)

Co—N1	2.1764 (19)
Co—N1*A*	2.110 (3)
Co—N1*B*	2.135 (3)

**Fig. 1.**
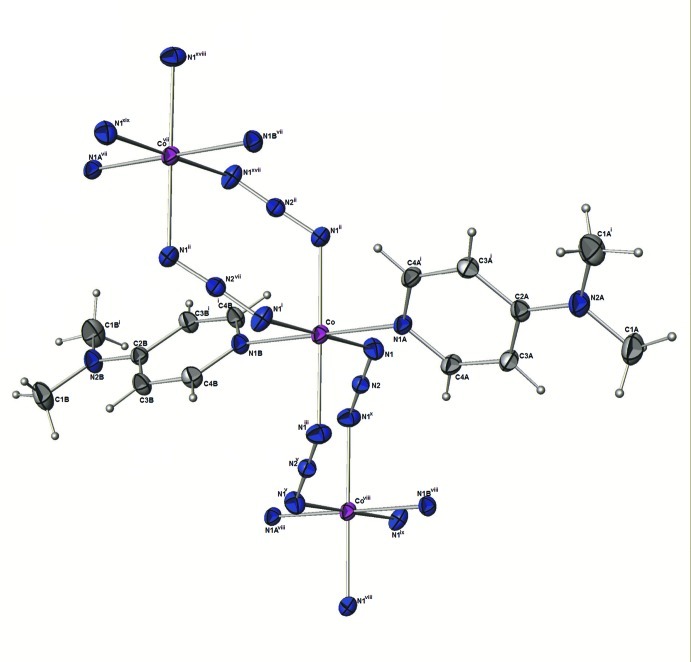
View of the structure of (I), showing the atom-numbering scheme. Displacement ellipsoids are drawn at the 50% probability level and H atoms are represented as spheres of arbitrary radii [symmetry codes: (i) x + 1, y, -z + 1/2; (ii) x, y, -z + 1/2; (iii) x + 1, y, z; (v) x + 1, -y + 1, -z; (vii) x + 1, -y + 1, z + 1/2; (viii) x + 1, -y + 1, z - 1/2; (ix) x, -y + 1, z - 1/2; (x) x, -y + 1, -z; (xvii) x, -y + 1, z + 1/2; (xviii) x, -y + 1, -z + 1; (xix) x + 1, -y + 1, -z + 1].

**Fig. 2.**
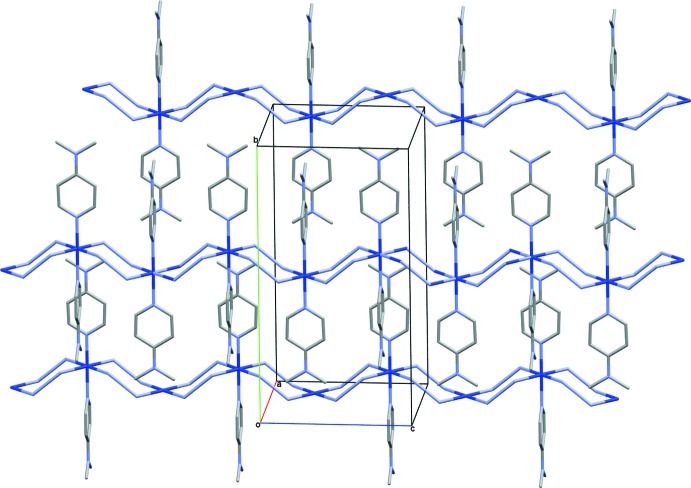
View of part of the crystal structure of (I), showing layers along the [001] direction. Hydrogen atoms are omitted for clarity.

**Fig. 3.**
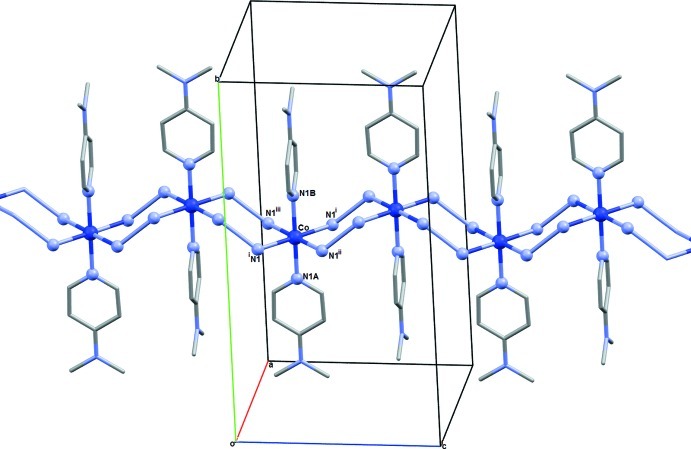
Part of the crystal structure, showing the octahedral coordination around the cobalt(II) atoms. Hydrogen atoms are omitted for clarity [symmetry codes: (i): -x + 1,y,-z + 1/2; (ii): x,y,-z + 1/2; (iii): -x + 1,y,z].

## supplementary crystallographic information

### Comment

The chemistry of coordination polymers has evolved rapidly in recent years and a variety of topologies has been constructed through ligand design and the use of different transition metal geometries. These polymers may have interesting properties and applications, *e.g.* adsorption, ion exchange, non-linear optical and magnetic materials (Hoskins *et al.*, 1990; Fujita *et al.*, 1994; Yaghi & Li, 1995; Hagrman *et al.*, 1999).

Pseudohalide anions are excellent ligands for obtaining discrete, one-dimensional, two-dimensional or three-dimensional systems. Among these, the azido ligand is the most versatile in linking divalent metal ions. When the azide group acts as bridging ligand there are two typical coordination modes: end-to-end (EE or µ-1,3) in which the resulting complexes usually shows ferromagnetic behavior, and end-on (EO or µ-1,1) which results in antiferromagnetic behavior.

In the course of our investigation of functional coordination complexes and polymers, a new azide-bridged coordination polymer with 4-dimethylaminopyridine has been prepared and structurally characterized.

Part of the structure of (I) with the atom numbering scheme is shown in Figure 1. The structure consists of layers of cobalt atoms linked by double end-to-end (EE) azido bridges, placed along the [001] direction at *b* = 0 and *b* = 1/2, forming a one-dimensional polymeric chain with each cobalt(II) ion in an octahedral environment (Fig. 2). In the crystal, parallel one-dimensional polymers form a three-dimensional network. The minimum interdinuclear Co···Co distance bridged by the EE-azido ligands is 5.097 (2) Å. In this structure, the ligand *L* displays monodentate binding to Co^II^.

The octahedral coordination around the cobalt(II) atoms (Fig. 3, Table 1) consists of two *L* ligands coordinated *via* the pyridine nitrogen atom which occupy the axial positions (Co—N1A = 2.110 (3) Å and Co—N1B = 2.135 (3) Å) and four azide bridges in the equatorial plane (Co—N1 = 2.1764 (19) Å) which act as symmetrical end-to-end (µ-1,3) double bridges betwee two neighboring cobalt atoms.

This structure can be compared with that observed for [Cu(*L*)2(N3)2]n (*L*: 4-dimethylaminopyridine (Dalai *et al.*, 2002), which shows also double end-to-end (EE) azido bridges. Here each copper is bonded to two nitrogen atoms of the pyridine ligands (1.999 (7) Å, 2.014 (7) Å) and two nitrogen atoms of the azide (2.029 (5) Å). There are also two weak attachments to two nitrogen atoms of the azide (2.611 (6) Å) in axial positions to create a doubl EE-bridged one-dimensional polymer with each copper(II) ion in a pseudo-octahedral environment. The distance between two neighboring copper ions is 5.20 (1) Å.

### Experimental

A mixture of NaN_3_ and CoCl_2_.6H_2_O in methanol was stirred for half an hour, then 4-dimethylaminopyridine was added to the solution and the reaction continued to stir for one hour. After filtration, the pink filtrate was allowed to stand at room temperature. Pink crystals were obtained by slow evaporation.

### Refinement

The aromatic H atoms were placed at calculated positions with C—H = 0.93 and 0.96 Å, for aromatic and methyl H atoms, respectively, with *U*_iso_(H) = 1.2*U*_eq_(C).

### Crystal data

**Table:**

[Co(N_3_)_2_(C_7_H_10_N_2_)_2_]	*F*(000) = 804
*M**_r_* = 387.33	*D*_x_ = 1.493 Mg m^−^^3^
Orthorhombic, *C**m**c**m*	Mo *K*α radiation, λ = 0.71073 Å
Hall symbol: -C 2c 2	Cell parameters from 1393 reflections
*a* = 9.622 (5) Å	θ = 3.1–30.0°
*b* = 18.404 (5) Å	µ = 1.02 mm^−^^1^
*c* = 9.734 (5) Å	*T* = 293 K
*V* = 1723.7 (13) Å^3^	Needle, pink
*Z* = 4	0.1 × 0.09 × 0.08 mm

### Data collection

**Table:**

Bruker APEXII diffractometer	1099 reflections with *I* > 2σ(*I*)
Radiation source: fine-focus sealed tube	*R*_int_ = 0.031
Graphite monochromator	θ_max_ = 30.0°, θ_min_ = 3.1°
φ scans	*h* = −11→13
5192 measured reflections	*k* = −25→25
1393 independent reflections	*l* = −9→13

### Refinement

**Table:**

Refinement on *F*^2^	0 restraints
Least-squares matrix: full	H-atom parameters constrained
*R*[*F*^2^ > 2σ(*F*^2^)] = 0.032	*w* = 1/[σ^2^(*F*_o_^2^) + (0.0383*P*)^2^ + 1.0521*P*] where *P* = (*F*_o_^2^ + 2*F*_c_^2^)/3
*wR*(*F*^2^) = 0.086	(Δ/σ)_max_ < 0.001
*S* = 1.07	Δρ_max_ = 0.43 e Å^−^^3^
1393 reflections	Δρ_min_ = −0.37 e Å^−^^3^
79 parameters	

### Special details

**Table:**

Geometry. Bond distances, angles *etc*. have been calculated using the rounded fractional coordinates. All su's are estimated from the variances of the (full) variance-covariance matrix. The cell e.s.d.'s are taken into account in the estimation of distances, angles and torsion angles
Refinement. Refinement of *F*^2^ against ALL reflections. The weighted *R*-factor *wR* and goodness of fit *S* are based on *F*^2^, conventional *R*-factors *R* are based on *F*, with *F* set to zero for negative *F*^2^. The threshold expression of *F*^2^ > σ(*F*^2^) is used only for calculating *R*-factors(gt) *etc*. and is not relevant to the choice of reflections for refinement. *R*-factors based on *F*^2^ are statistically about twice as large as those based on *F*, and *R*- factors based on ALL data will be even larger.

### Fractional atomic coordinates and isotropic or equivalent isotropic displacement parameters (Å^2^)

**Table:**

	*x*	*y*	*z*	*U*_iso_*/*U*_eq_	Occ. (<1)
Co	0.50000	0.45887 (2)	0.25000	0.0257 (1)	
N1	0.33908 (16)	0.45953 (7)	0.09288 (16)	0.0355 (4)	
N1A	0.50000	0.34420 (13)	0.25000	0.0267 (8)	
N1B	0.50000	0.57488 (14)	0.25000	0.0279 (8)	
N2	0.34145 (19)	0.50000	0.00000	0.0263 (5)	
N2A	0.50000	0.11621 (15)	0.25000	0.0383 (10)	
N2B	0.50000	0.80246 (14)	0.25000	0.0358 (9)	
C1A	0.50000	0.07558 (14)	0.1240 (3)	0.0536 (10)	
C1B	0.6285 (3)	0.84282 (14)	0.25000	0.0514 (9)	
C2A	0.50000	0.19006 (16)	0.25000	0.0280 (9)	
C2B	0.50000	0.72899 (16)	0.25000	0.0264 (9)	
C3A	0.50000	0.23072 (12)	0.1278 (3)	0.0315 (7)	
C3B	0.6237 (2)	0.68797 (12)	0.25000	0.0318 (7)	
C4A	0.50000	0.30567 (12)	0.1330 (3)	0.0311 (7)	
C4B	0.6178 (2)	0.61381 (12)	0.25000	0.0313 (7)	
H1B1	0.60862	0.89393	0.25000	0.0769*	
H1B2	0.68126	0.83069	0.16947	0.0769*	0.500
H1B3	0.68126	0.83069	0.33053	0.0769*	0.500
H3A	0.50000	0.20711	0.04332	0.0377*	
H3B	0.70933	0.71138	0.25000	0.0382*	
H1A1	0.50000	0.02454	0.14436	0.0805*	
H4A	0.50000	0.33101	0.05031	0.0373*	
H4B	0.70142	0.58851	0.25000	0.0376*	
H1A2	0.58146	0.08752	0.07175	0.0805*	0.500
H1A3	0.41854	0.08752	0.07175	0.0805*	0.500

### Atomic displacement parameters (Å^2^)

**Table:**

	*U*^11^	*U*^22^	*U*^33^	*U*^12^	*U*^13^	*U*^23^
Co	0.0356 (3)	0.0196 (2)	0.0218 (2)	0.0000	0.0000	0.0000
N1	0.0437 (8)	0.0333 (7)	0.0296 (8)	−0.0070 (6)	−0.0062 (7)	0.0078 (6)
N1A	0.0357 (15)	0.0223 (11)	0.0222 (14)	0.0000	0.0000	0.0000
N1B	0.0291 (14)	0.0221 (12)	0.0324 (16)	0.0000	0.0000	0.0000
N2	0.0259 (9)	0.0259 (8)	0.0270 (10)	0.0000	0.0000	−0.0017 (8)
N2A	0.0530 (19)	0.0234 (13)	0.0385 (18)	0.0000	0.0000	0.0000
N2B	0.0355 (15)	0.0209 (12)	0.051 (2)	0.0000	0.0000	0.0000
C1A	0.074 (2)	0.0299 (13)	0.057 (2)	0.0000	0.0000	−0.0100 (13)
C1B	0.0459 (16)	0.0292 (12)	0.079 (2)	−0.0094 (11)	0.0000	0.0000
C2A	0.0265 (15)	0.0234 (13)	0.0341 (19)	0.0000	0.0000	0.0000
C2B	0.0277 (15)	0.0242 (13)	0.0272 (17)	0.0000	0.0000	0.0000
C3A	0.0422 (13)	0.0266 (10)	0.0256 (12)	0.0000	0.0000	−0.0049 (9)
C3B	0.0227 (10)	0.0283 (10)	0.0445 (15)	−0.0022 (8)	0.0000	0.0000
C4A	0.0450 (13)	0.0274 (10)	0.0209 (12)	0.0000	0.0000	0.0016 (9)
C4B	0.0242 (11)	0.0287 (10)	0.0411 (14)	0.0036 (8)	0.0000	0.0000

### Geometric parameters (Å, º)

**Table:**

Co—N1	2.1764 (19)	C2A—C3A	1.405 (3)
Co—N1A	2.110 (3)	C2A—C3A^i^	1.405 (3)
Co—N1B	2.135 (3)	C2B—C3B	1.410 (3)
Co—N1^i^	2.1764 (19)	C2B—C3B^i^	1.410 (3)
Co—N1^ii^	2.1764 (19)	C3A—C4A	1.380 (3)
Co—N1^iii^	2.1764 (19)	C3B—C4B	1.366 (3)
N1—N2	1.1716 (16)	C1A—H1A1	0.9600
N1A—C4A	1.342 (3)	C1A—H1A2	0.9600
N1A—C4A^i^	1.342 (3)	C1A—H1A3	0.9600
N1B—C4B	1.341 (3)	C1B—H1B1	0.9600
N1B—C4B^i^	1.340 (3)	C1B—H1B2	0.9600
N2A—C1A	1.437 (3)	C1B—H1B3	0.9600
N2A—C2A	1.359 (4)	C3A—H3A	0.9300
N2A—C1A^i^	1.437 (3)	C3B—H3B	0.9300
N2B—C1B	1.442 (3)	C4A—H4A	0.9300
N2B—C2B	1.352 (4)	C4B—H4B	0.9300
N2B—C1B^i^	1.442 (3)		
			
N1—Co—N1A	90.32 (4)	N2A—C2A—C3A^i^	122.17 (14)
N1—Co—N1B	89.68 (4)	C3A—C2A—C3A^i^	115.7 (2)
N1—Co—N1^i^	179.36 (5)	N2B—C2B—C3B	122.39 (13)
N1—Co—N1^ii^	89.29 (6)	N2B—C2B—C3B^i^	122.39 (13)
N1—Co—N1^iii^	90.70 (6)	C3B—C2B—C3B^i^	115.2 (2)
N1A—Co—N1B	180.00	C2A—C3A—C4A	120.1 (3)
N1^i^—Co—N1A	90.32 (4)	C2B—C3B—C4B	120.00 (19)
N1^ii^—Co—N1A	90.32 (4)	N1A—C4A—C3A	124.0 (3)
N1^iii^—Co—N1A	90.32 (4)	N1B—C4B—C3B	124.68 (19)
N1^i^—Co—N1B	89.68 (4)	N2A—C1A—H1A1	109.00
N1^ii^—Co—N1B	89.68 (4)	N2A—C1A—H1A2	109.00
N1^iii^—Co—N1B	89.68 (4)	N2A—C1A—H1A3	109.00
N1^i^—Co—N1^ii^	90.70 (6)	H1A1—C1A—H1A2	109.00
N1^i^—Co—N1^iii^	89.29 (6)	H1A1—C1A—H1A3	109.00
N1^ii^—Co—N1^iii^	179.36 (5)	H1A2—C1A—H1A3	109.00
Co—N1—N2	122.14 (12)	N2B—C1B—H1B1	110.00
Co—N1A—C4A	121.91 (14)	N2B—C1B—H1B2	109.00
Co—N1A—C4A^i^	121.91 (14)	N2B—C1B—H1B3	109.00
C4A—N1A—C4A^i^	116.2 (2)	H1B1—C1B—H1B2	109.00
Co—N1B—C4B	122.30 (13)	H1B1—C1B—H1B3	109.00
Co—N1B—C4B^i^	122.32 (13)	H1B2—C1B—H1B3	109.00
C4B—N1B—C4B^i^	115.4 (2)	C2A—C3A—H3A	120.00
N1—N2—N1^iv^	177.8 (2)	C4A—C3A—H3A	120.00
C1A—N2A—C2A	121.37 (14)	C2B—C3B—H3B	120.00
C1A—N2A—C1A^i^	117.3 (2)	C4B—C3B—H3B	120.00
C1A^i^—N2A—C2A	121.37 (14)	N1A—C4A—H4A	118.00
C1B—N2B—C2B	121.00 (14)	C3A—C4A—H4A	118.00
C1B—N2B—C1B^i^	118.0 (2)	N1B—C4B—H4B	118.00
C1B^i^—N2B—C2B	121.00 (14)	C3B—C4B—H4B	118.00
N2A—C2A—C3A	122.17 (14)		

Symmetry codes: (i) −*x*+1, *y*, −*z*+1/2; (ii) *x*, *y*, −*z*+1/2; (iii) −*x*+1, *y*, *z*; (iv) *x*, −*y*+1, −*z*.
